# Emergence of the FLAIR Ivy Sign During Progressive Posterior Cerebral Artery Occlusion and Postoperative Disappearance After Revascularization Surgery in Adult Moyamoya Disease: A Case Report

**DOI:** 10.1155/crra/1597887

**Published:** 2026-11-10

**Authors:** Shunji Mugikura, Naoko Mori

**Affiliations:** ^1^ Department of Diagnostic Radiology Graduate School of Medicine Tohoku University Sendai Japan tohoku.ac.jp; ^2^ Department of Radiology Graduate School of Medicine Akita University Akita Japan akita-u.ac.jp

**Keywords:** case report, cerebral revascularization, FLAIR ivy sign, moyamoya disease, posterior cerebral artery

## Abstract

The FLAIR ivy sign is a characteristic MRI finding in moyamoya disease and is thought to reflect impaired cerebrovascular reserve. However, its longitudinal relationship with leptomeningeal collateral failure associated with progressive posterior cerebral artery (PCA) involvement remains incompletely understood. A woman in her 40s with moyamoya disease had undergone left superficial temporal artery–middle cerebral artery (STA–MCA) bypass surgery for left hemispheric ischemia 10 years earlier and remained neurologically stable during long‐term follow‐up. During a stable postoperative phase, follow‐up magnetic resonance angiography (MRA) demonstrated mild narrowing of the proximal right PCA with preserved visualization of the distal PCA branches, suggesting maintained PCA‐derived leptomeningeal collateral circulation. Approximately 6 months later, the patient developed recurrent transient ischemic attacks involving the right cerebral hemisphere, accompanied by progression to complete occlusion of the right PCA. At this symptomatic preoperative stage, FLAIR MRI demonstrated a newly developed ivy sign involving both the anterior and posterior portions of the right MCA territory, and diffusion‐weighted imaging demonstrated multiple acute infarctions predominantly involving the posterior portion of the MCA territory. Right STA–MCA bypass surgery was subsequently performed. In the acute postoperative phase, focal hyperperfusion accompanied by localized convexity subarachnoid hemorrhage developed at the frontal STA–MCA bypass recipient site. During chronic postoperative follow‐up, the patient’s ischemic symptoms resolved completely, and follow‐up MRI demonstrated complete disappearance of the ivy sign. This case suggests that progressive impairment of previously preserved PCA‐derived leptomeningeal collaterals may result in widespread hemodynamic compromise within the MCA territory, manifested as a newly developed FLAIR ivy sign. Although postoperative hyperperfusion and acute infarction represented distinct hemodynamic consequences, longitudinal evaluation of the FLAIR ivy sign may provide useful insight into ischemic progression, impaired cerebrovascular reserve, and perioperative hemodynamic changes in moyamoya disease.

## 1. Introduction

Moyamoya disease is a chronic progressive cerebrovascular disorder characterized by steno‐occlusive changes involving the terminal portions of the internal carotid arteries (ICAs) and the development of compensatory collateral networks [1, 2]. As the disease progresses, steno‐occlusive involvement may extend to the posterior cerebral artery (PCA), potentially compromising PCA‐derived collateral perfusion to the anterior circulation [3, 4].

The fluid‐attenuated inversion recovery (FLAIR) ivy sign is a characteristic imaging finding in moyamoya disease, appearing as linear or curvilinear hyperintensities along the cortical sulci [5, 6]. The ivy sign has been associated with impaired cerebrovascular reserve and is generally considered to reflect slow flow within dilated leptomeningeal vessels in the setting of chronic hemodynamic compromise [5, 6]. Longitudinal evaluation of the ivy sign may therefore provide insight into changes in cerebral hemodynamic status in moyamoya disease. However, the longitudinal relationship between progressive PCA involvement, impairment of PCA‐derived leptomeningeal collateral circulation, and temporal changes in the FLAIR ivy sign remains incompletely understood.

Here, we report an adult patient with moyamoya disease who developed unilateral PCA occlusion after a prolonged period of clinical stability. Serial MRI documented the transition from mild PCA stenosis without an ivy sign to PCA occlusion accompanied by newly developed ivy signs, followed by their disappearance after revascularization. Our previous report described the concept of ischemic territorial mismatch associated with PCA occlusion [7]. The present case extends that observation by demonstrating a within‐patient hemispheric contrast between PCA‐related collateral failure and compensated ICA progression, together with the spatial dissociation among the widespread FLAIR ivy sign, posterior MCA infarction, and focal anterior MCA hyperperfusion after bypass surgery.

## 2. Case Report

The clinical course is summarized in Table 1 and can be broadly divided into four hemodynamic phases (Figure 1). A woman in her 40s had presented with recurrent transient ischemic attacks (TIAs) involving the left cerebral hemisphere 10 years earlier and was diagnosed with moyamoya disease based on bilateral steno‐occlusive changes of the ICAs with abnormal basal collateral vessels. She subsequently underwent left superficial temporal artery–middle cerebral artery (STA–MCA) bypass surgery with additional indirect revascularization for left hemispheric ischemia. At that time, no revascularization procedure was performed for the right cerebral hemisphere because it was asymptomatic. After surgery, her ischemic symptoms resolved completely, and she remained neurologically stable during long‐term follow‐up. She had a medical history of obesity, borderline diabetes mellitus, and dyslipidemia.

**Table 1:** Timeline of the clinical and imaging course according to the proposed four‐phase hemodynamic framework.

Clinical phase	Time	Clinical course	Imaging findings	Management	Figure
Initial presentation	10 years before current admission	Recurrent left hemispheric TIAs	Bilateral moyamoya disease	Left STA–MCA bypass + EDMS	—
Compensated collateral phase	9 years after left bypass	Neurologically stable	Patent left bypass; mild right PCA stenosis; no FLAIR ivy sign	Observation	Figure 2
Symptomatic phase associated with progressive PCA occlusion	Current admission, approximately 6 months after previous MRI	Recurrent right hemispheric TIAs	Right PCA occlusion; FLAIR ivy sign in the anterior and posterior MCA territories; posterior MCA infarctions; reduced PCA‐derived superficial collaterals	Medical therapy → right STA–MCA bypass + EDMS	Figures 3, 4, and 5
Early postoperative hyperperfusion phase	Postoperative Day 2	No new neurological deficits	Focal hyperperfusion at the frontal STA–MCA bypass recipient site on ^123^ I‐IMP SPECT; localized convexity SAH	Strict blood pressure control	Figure 6
Chronic postoperative recovery phase	5 months after surgery	No recurrent TIAs	Patent bypass; resolution of the FLAIR ivy sign	Follow‐up	Figure 7
Long‐term follow‐up	5 years after surgery	Neurologically stable; no recurrent cerebrovascular events	—	Long‐term follow‐up	—

*Note:* The clinical phases correspond to the conceptual four‐phase hemodynamic framework illustrated in Figure 1, whereas the figure citations in the table refer to the actual imaging findings presented in Figures 2, 3, 4, 5, 6, and 7.

Abbreviations: ACA, anterior cerebral artery; DSA, digital subtraction angiography; EDMS, encephalo‐duro‐myo‐synangiosis; FLAIR, fluid‐attenuated inversion recovery; MCA, middle cerebral artery; MRA, magnetic resonance angiography; PCA, posterior cerebral artery; POD, postoperative day; SPECT, single‐photon emission computed tomography; STA–MCA, superficial temporal artery–middle cerebral artery; TIA, transient ischemic attack.

**Figure 1:**
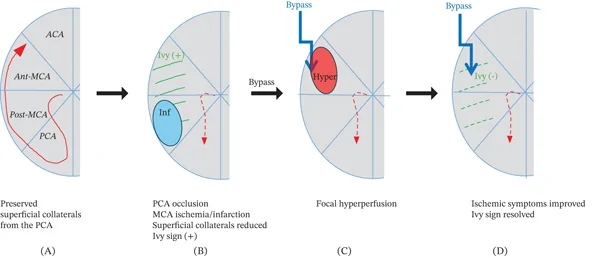
Schematic summary of the longitudinal hemodynamic changes associated with the FLAIR ivy sign in the present case. A woman in her 40s with moyamoya disease had previously undergone contralateral superficial temporal artery–middle cerebral artery (STA–MCA) bypass surgery and remained neurologically stable during long‐term follow‐up. This schematic illustrates the proposed hemodynamic changes associated with progressive posterior cerebral artery (PCA) steno‐occlusive disease and the development of the FLAIR ivy sign. It focuses on PCA‐derived superficial leptomeningeal collateral vessels supplying the middle cerebral artery (MCA) territory (“collaterals” in this figure). Red solid and dashed lines represent preserved and reduced PCA‐derived superficial leptomeningeal collateral flow, respectively. Green solid and dashed lines indicate the presence and postoperative resolution of the FLAIR ivy sign, respectively. Blue and red ovals represent acute infarction and focal postoperative hyperperfusion, respectively, while blue lines indicate STA–MCA bypass flow. In the present case, progressive PCA steno‐occlusive changes were associated with widespread hemodynamic compromise within the MCA territory, manifested by a newly developed FLAIR ivy sign involving both the anterior and posterior MCA territories. Although both territories may be affected, the posterior MCA territory is presumed to rely more heavily on PCA‐derived superficial leptomeningeal collateral circulation than the anterior MCA territory because of its greater distance from the anterior circulation, and may therefore be more susceptible to the hemodynamic consequences of progressive PCA steno‐occlusive changes. Consistent with this concept, acute infarction occurred in the posterior MCA territory. In contrast, postoperative hyperperfusion developed at the frontal STA–MCA bypass recipient site in the anterior MCA territory, whereas the preoperative FLAIR ivy sign involved both the anterior and posterior MCA territories. This schematic was adapted from the conceptual framework of ischemic territorial mismatch associated with PCA‐related collateral insufficiency in moyamoya disease previously proposed by our group [7]. (A) Compensated collateral phase. During a stable postoperative phase after contralateral bypass surgery, preserved collateral circulation from the posterior cerebral artery (PCA) supplies the anterior circulation. Distal PCA‐derived superficial leptomeningeal collaterals remain well developed, maintaining adequate cerebrovascular reserve in the middle cerebral artery (MCA) territory. No transient ischemic attacks (TIAs) occur, and no FLAIR ivy sign is observed. (B) Symptomatic phase associated with progressive PCA occlusion. With progression from mild stenosis to complete occlusion of the posterior cerebral artery (PCA), collateral flow from the PCA to the anterior circulation decreases markedly. Impairment of these superficial leptomeningeal collaterals is associated with reduced cerebrovascular reserve and the development of transient ischemic attacks (TIAs). The FLAIR ivy sign emerges predominantly in the middle cerebral artery (MCA) territory and may reflect chronic hemodynamic compromise associated with PCA‐related collateral insufficiency. (C) Early postoperative hyperperfusion phase. Following right STA–MCA bypass surgery, focal postoperative hyperperfusion develops in the anterior MCA territory, corresponding to the bypass recipient site. Although the preoperative FLAIR ivy sign involved both the anterior and posterior MCA territories, postoperative hyperperfusion is localized to the bypass territory and should be distinguished from the posterior ischemic territory. (D) Chronic postoperative recovery phase. In the chronic postoperative phase, cerebral hemodynamics stabilize as collateral circulation is reconstituted and perfusion reserve is restored. Symptoms of ischemia, including TIAs, resolve, and the previously observed FLAIR ivy sign disappear, reflecting recovery from chronic hemodynamic compromise.

The clinical and hemodynamic course of the present case could be broadly divided into four phases: a compensated collateral phase with preserved PCA‐derived collateral supply to the anterior circulation (Figure 1A), a symptomatic phase associated with progressive PCA occlusion (Figure 1B), an early postoperative hyperperfusion phase after revascularization surgery (Figure 1C), and a chronic postoperative recovery phase (Figure 1D) [7].

MRI examinations were performed using a 3.0‐T scanner (Achieva, Philips Healthcare, Best, The Netherlands). FLAIR images were acquired with the following parameters: repetition time (TR), 11,000 ms; echo time (TE), 125 ms; inversion time (TI), 2800 ms; flip angle, 90°; matrix, 210 × 210; and slice thickness, 6 mm. Time‐of‐flight magnetic resonance angiography (TOF‐MRA) was obtained with TR of 25 ms, TE of 3.453 ms, flip angle of 20°, slice thickness of 1.4 mm, matrix of 210 × 200, and 180 slices.

### 2.1. Compensated Collateral Phase (Figure 1A)

Nine years after the initial left‐sided bypass surgery, follow‐up magnetic resonance angiography (MRA) demonstrated good patency of the left STA–MCA bypass graft with preserved extracranial‐to‐intracranial collateral flow supplying the left cerebral hemisphere (Figure 2A). Mild stenosis was observed in the proximal portion of the right PCA, although distal PCA branches remained well visualized, suggesting preserved PCA‐derived superficial leptomeningeal collateral supply to the anterior circulation. In contrast, no significant steno‐occlusive changes were observed in the left PCA.

**Figure 2:**
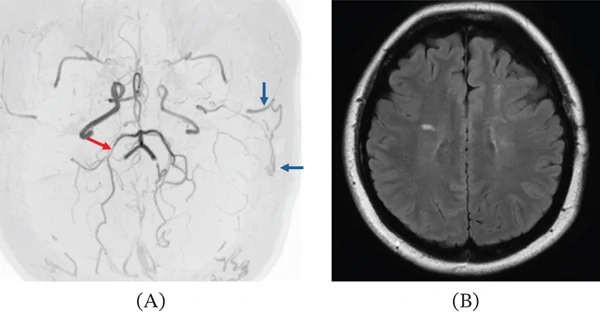
Imaging findings during the compensated collateral phase. A woman in her 40s with moyamoya disease had previously undergone left superficial temporal artery–middle cerebral artery (STA–MCA) bypass surgery with additional indirect revascularization for ischemic symptoms involving the left cerebral hemisphere and remained neurologically stable during long‐term follow‐up. (A) Axial magnetic resonance angiography (MRA) obtained 9 years after left‐sided bypass surgery demonstrates bilateral steno‐occlusive changes of the internal carotid arteries (ICAs), consistent with moyamoya disease. The left STA–MCA bypass graft is patent, and extracranial‐to‐intracranial collateral flow supplying the left cerebral hemisphere is well preserved (blue arrows). Mild narrowing is observed in the proximal portion of the right posterior cerebral artery (PCA) (red arrow), while distal PCA branches remain well visualized, suggesting preserved PCA–derived leptomeningeal collateral supply to the anterior circulation. No significant steno‐occlusive changes are observed in the left PCA. (B) Axial fluid‐attenuated inversion recovery (FLAIR) imaging obtained at the same examination demonstrates no FLAIR ivy sign in either cerebral hemisphere. Old ischemic lesions are observed predominantly in the bilateral periventricular deep white matter, consistent with previous ischemic injury. Diffusion‐weighted imaging showed no acute ischemic lesions (not shown).

FLAIR imaging obtained at the same examination demonstrated no ivy sign in either cerebral hemisphere (Figure 2B). Old ischemic lesions were observed predominantly in the bilateral periventricular deep white matter, consistent with previous ischemic injury, whereas diffusion‐weighted imaging showed no acute ischemic lesions (not shown).

During this stable follow‐up period, the patient remained free of recurrent ischemic symptoms. Steno‐occlusive changes involving the right ICA system showed no significant progression on MRA, and collateral circulation via the previous left‐sided bypass remained well preserved.

### 2.2. Symptomatic Phase Associated With Progressive PCA Occlusion (Figure 1B)

Approximately 6 months after the stable follow‐up examination shown in Figure 2, the patient developed recurrent episodes of transient numbness involving the left face and upper extremity without persistent neurological deficits, consistent with recurrent TIAs involving the right cerebral hemisphere. MRA obtained during this symptomatic phase demonstrated progression from mild proximal stenosis to complete occlusion of the right PCA (Figure 3A). Diffusion‐weighted imaging obtained during this symptomatic phase demonstrated multiple acute ischemic foci in the posterior portion of the right MCA territory, predominantly involving the parietal lobe (Figure 4). The patient was treated with intravenous edaravone and ozagrel sodium while awaiting surgical revascularization. During this period, the ischemic symptoms stabilized. Mild blood pressure control was achieved with oral candesartan before surgical revascularization.

**Figure 3:**
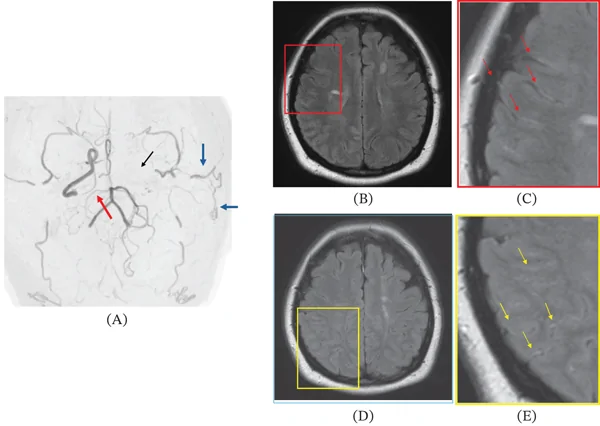
MRA and FLAIR imaging during the symptomatic phase associated with progressive PCA occlusion. (A) Axial magnetic resonance angiography (MRA) obtained at the onset of frequent right hemispheric transient ischemic attacks demonstrates occlusion of the right posterior cerebral artery (PCA) (red arrow). This artery had appeared mildly narrowed on MRA obtained during the previous stable follow‐up examination (Figure 2A). Notably, in the contralateral left hemisphere, which remained asymptomatic, the internal carotid artery (ICA), previously visualized on the prior examination, was no longer identifiable (black arrow). Despite this progression, extracranial‐to‐intracranial collateral pathways established by the previous bypass remained well preserved (blue arrows). In addition, the left PCA showed no steno‐occlusive changes and was presumed to continue providing leptomeningeal collateral flow to the anterior circulation. (B, C) Axial fluid‐attenuated inversion recovery (FLAIR) images demonstrate a newly developed ivy sign involving the anterior portion of the right MCA territory. Panel (B) shows the original image, and panel (C) is a magnified view of the boxed region in (B). Linear or curvilinear cortical hyperintensities along the cerebral sulci (red arrows) represent the FLAIR ivy sign. (D, E) Axial FLAIR images demonstrate a newly developed ivy sign involving the posterior portion of the right MCA territory, including the parietal region where acute infarctions are identified on diffusion‐weighted imaging (Figure 4). Panel (D) shows the original image, and panel (E) is a magnified view of the boxed region in (D). Linear or curvilinear cortical hyperintensities along the cerebral sulci (yellow arrows) represent the FLAIR ivy sign.

**Figure 4:**
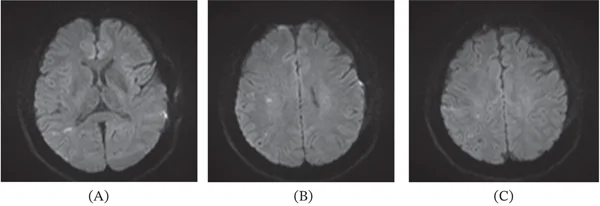
(A–C) Diffusion‐weighted imaging during the symptomatic phase associated with progressive PCA occlusion. Diffusion‐weighted imaging obtained during the same preoperative symptomatic phase as Figure 3 demonstrates multiple acute ischemic infarctions predominantly involving the posterior portion of the right MCA territory, including the parietal lobe. The distribution of the lesions is consistent with preferential involvement of the posterior aspect of the MCA territory in association with progressive PCA steno‐occlusive changes.

Catheter angiography performed during the same preoperative period further demonstrated the characteristic hemodynamic features of moyamoya disease (Figure 5). Right internal carotid angiography demonstrated angiographic findings corresponding to Suzuki stage III, characterized by terminal ICA steno‐occlusive changes, prominent basal moyamoya vessels, and markedly reduced antegrade filling of the right MCA territory (Figure 5A). Left internal carotid angiography demonstrated angiographic findings corresponding to Suzuki Stage VI, characterized by disappearance of the basal moyamoya vessels and no appreciable antegrade visualization of the intracranial ICA circulation (Figure 5B).

**Figure 5:**
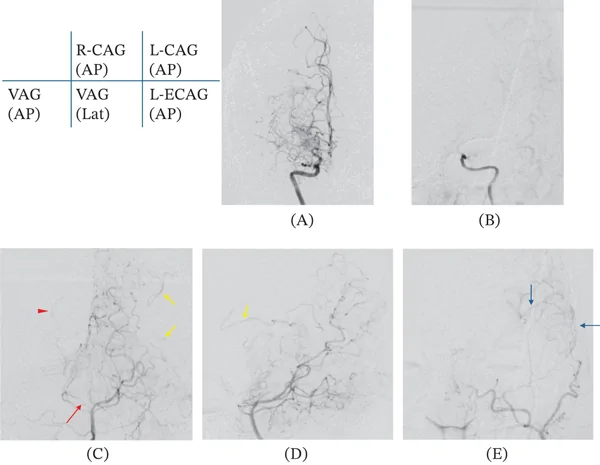
Preoperative digital subtraction angiography (DSA) during the symptomatic phase associated with progressive PCA occlusion. Abbreviations: ACA, anterior cerebral artery; AP, anteroposterior view; CAG, carotid angiography; DSA, digital subtraction angiography; ECAG, external carotid angiography; ICA, internal carotid artery; Lat, lateral view; MCA, middle cerebral artery; PCA, posterior cerebral artery; VAG, vertebral angiography. (A) Right internal carotid DSA demonstrates angiographic findings corresponding to Suzuki stage III, characterized by terminal internal carotid artery steno‐occlusive changes, prominent basal moyamoya vessels, and markedly reduced antegrade filling of the right middle cerebral artery (MCA) territory. (B) Left internal carotid DSA demonstrates angiographic findings corresponding to Suzuki stage VI, characterized by disappearance of the basal moyamoya vessels and no appreciable antegrade visualization of the intracranial internal carotid artery (ICA) circulation. (C) Anteroposterior vertebral DSA demonstrates severe steno‐occlusive involvement at the origin of the right posterior cerebral artery (PCA) (red arrow). Although faint antegrade opacification of the distal right PCA is preserved (red arrowhead), PCA‐derived superficial leptomeningeal collateral vessels supplying the right MCA territory are markedly reduced compared with the contralateral side. In contrast, the left PCA shows no steno‐occlusive changes and gives rise to well‐developed PCA‐derived superficial leptomeningeal collateral vessels supplying the left MCA territory (yellow arrows). (D) Vertebral DSA demonstrates a patent distal PCA, most likely representing the left PCA, giving rise to well‐developed superficial leptomeningeal collateral vessels. Deep leptomeningeal collateral vessels supplying the anterior cerebral artery (ACA) territory via the posterior pericallosal artery are also clearly visualized (yellow arrow). (E) Left external carotid DSA demonstrates a patent superficial temporal artery–middle cerebral artery (STA–MCA) bypass with well‐developed extracranial‐to‐intracranial collateral circulation supplying the left MCA territory (blue arrows).

Anteroposterior vertebral angiography revealed severe steno‐occlusive involvement at the origin of the right PCA. Although faint antegrade opacification of the distal right PCA was preserved, PCA‐derived superficial leptomeningeal collateral vessels supplying the right MCA territory were markedly reduced. In contrast, the left PCA showed no steno‐occlusive changes and gave rise to well‐developed PCA‐derived superficial leptomeningeal collateral vessels supplying the left MCA territory (Figure 5C).

Lateral vertebral angiography demonstrated a patent distal PCA, most likely representing the left PCA, giving rise to well‐developed superficial leptomeningeal collateral vessels. Deep leptomeningeal collateral vessels supplying the anterior cerebral artery (ACA) territory via the posterior pericallosal artery were also clearly visualized (Figure 5D).

Left external carotid angiography demonstrated a widely patent STA–MCA bypass with robust extracranial‐to‐intracranial collateral flow supplying the left MCA territory (Figure 5E).

Concurrent FLAIR imaging revealed a newly developed ivy sign involving both the anterior and posterior portions of the right MCA territory (Figure 3B–E). The ivy sign was observed in the frontal MCA territory as well as in the posterior MCA territory, including the parietal lobe where acute infarctions were identified on diffusion‐weighted imaging (Figure 4). In contrast, in the left hemisphere where prior revascularization had been performed, extracranial‐to‐intracranial collateral circulation remained well preserved on MRA (Figure 3A, blue arrows). Notably, the left ICA, which had been visualized at the previous follow‐up examination (Figure 2A), was no longer identifiable during this symptomatic phase (Figure 3A, black arrow). Nevertheless, no ischemic symptoms or FLAIR ivy sign were observed in the left hemisphere (Figure 3B,D). The left PCA also remained patent without occlusive changes. These imaging findings were temporally associated with the frequent right hemispheric TIAs, suggesting progressive hemodynamic compromise with resultant acute infarctions.

### 2.3. Early Postoperative Hyperperfusion Phase (Figure 1C)

Because of progressive clinical deterioration and imaging findings suggestive of hemodynamic compromise, right STA–MCA bypass combined with encephalo‐duro‐myo‐synangiosis (EDMS) was performed. The frontal branch of the STA was anastomosed to a 0.8‐mm M4 cortical branch supplying the right frontal lobe, with a temporary arterial occlusion time of 23 min. Marked dilatation of cortical vessels was observed intraoperatively.

Cerebral perfusion was evaluated using ^123^I‐N‐isopropyl‐p‐iodoamphetamine (^123^I‐IMP) single‐photon emission computed tomography (SPECT) (Symbia, Siemens Healthineers, Erlangen, Germany) after intravenous administration of 111 MBq of ^123^I‐IMP. Preoperative ^123^I‐IMP SPECT demonstrated bilateral hypoperfusion predominantly involving the MCA territories (Figure 6A). Postoperatively, systolic blood pressure was maintained below 140 mmHg, and no new neurological deficits developed after surgery.

**Figure 6:**
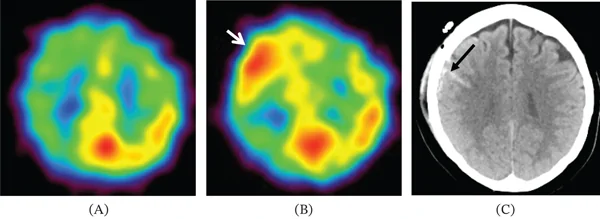
Preoperative and acute postoperative SPECT and CT findings. (A) Preoperative ^123^I‐IMP single‐photon emission computed tomography (SPECT) demonstrates bilateral hypoperfusion predominantly involving the middle cerebral artery (MCA) territory. (B) SPECT obtained on postoperative Day 2 reveals focal hyperperfusion in the anterior portion of the right MCA territory corresponding to the bypassed region (arrow). (C) Noncontrast computed tomography obtained during the early postoperative period demonstrates localized convexity subarachnoid hemorrhage (arrow). ^123^I‐IMP, iodine‐123 N‐isopropyl‐p‐iodoamphetamine.

On postoperative Day 2, follow‐up SPECT revealed focal hyperperfusion in the anterior portion of the right MCA territory corresponding to the bypassed region (Figure 6B). Postoperative cerebral hyperperfusion was diagnosed according to previously reported criteria [8], based on focal hyperperfusion on postoperative 123I‐IMP SPECT corresponding to the bypass territory, confirmation of bypass patency on MRA, and exclusion of alternative causes such as cerebral infarction on magnetic resonance imaging. Noncontrast computed tomography performed on the same day demonstrated localized convexity subarachnoid hemorrhage adjacent to the bypass site (Figure 6C). Blood pressure control was subsequently intensified to maintain systolic blood pressure below 130 mmHg. The patient remained neurologically stable thereafter.

### 2.4. Chronic Postoperative Recovery Phase (Figure 1D)

At 5 months after right‐sided bypass surgery, follow‐up MRA demonstrated good patency of the bypass graft with well‐established collateral flow (Figure 7A). Clinically, the patient remained neurologically intact without recurrent TIAs or other ischemic symptoms. Follow‐up FLAIR imaging demonstrated complete disappearance of the previously observed ivy sign in the right cerebral hemisphere (Figure 7B,C). During the subsequent 5‐year clinical follow‐up, no recurrent TIAs, cerebral infarction, or hemorrhagic cerebrovascular events occurred, and the patient remained neurologically stable.

**Figure 7:**
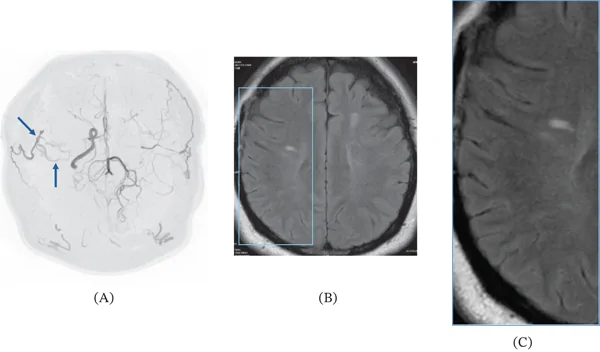
Imaging findings during the chronic postoperative recovery phase. (A) Magnetic resonance angiography (MRA) obtained 5 months after right‐sided bypass surgery demonstrates good patency of the superficial temporal artery–middle cerebral artery (STA–MCA) bypass with well‐established extracranial‐to‐intracranial collateral flow (blue arrows). (B) Axial fluid‐attenuated inversion recovery (FLAIR) image obtained at the same postoperative examination demonstrates complete disappearance of the previously observed FLAIR ivy sign (Figure 3) in the right cerebral hemisphere. The blue box indicates the region enlarged in (C). (C) Magnified view of the boxed region in (B) confirms complete resolution of the previously observed FLAIR ivy sign, with no residual cortical or sulcal hyperintensity.

## 3. Discussion

The principal observation in this case was the temporal and hemispheric correspondence between loss of PCA‐derived collateral support and changes in the FLAIR ivy sign. On the right, progression from mild PCA stenosis to occlusion coincided with recurrent ischemic symptoms, newly visible ivy signs, and posterior MCA infarction. On the left, by contrast, progression of ICA disease was not accompanied by either an ivy sign or recurrent ischemic symptoms in the presence of a patent bypass and preserved PCA collateral supply. This within‐patient contrast suggests that the change in the ivy sign was more closely related to deterioration of collateral support than to progression of ICA disease alone.

The FLAIR ivy sign has been associated with impaired cerebral perfusion and reduced cerebrovascular reserve in moyamoya disease [5, 6]. In the present case, its distribution was broader than that of the acute infarctions: the ivy sign involved both anterior and posterior MCA territories, whereas acute infarctions were concentrated posteriorly. This spatial difference suggests that the ivy sign reflected a broader zone of hemodynamic stress rather than the eventual distribution of irreversible ischemic injury.

One possible explanation for the predominant posterior MCA infarction in the present case is as follows. In moyamoya disease, PCA‐derived collateral pathways to the anterior circulation include superficial collateral pathways mainly supplying the MCA territory and deeper collateral pathways toward the ACA territory via the posterior pericallosal artery. In the present case, we speculate that the posterior MCA territory may have depended more heavily on PCA‐derived superficial leptomeningeal collateral flow than the anterior MCA territory because of its greater distance from the anterior circulation. Progressive PCA steno‐occlusive changes may therefore have preferentially compromised collateral perfusion to the posterior MCA territory, providing a possible explanation for the posterior MCA infarction despite the more widespread distribution of the FLAIR ivy sign involving both the anterior and posterior MCA territories. This interpretation is also consistent with the recently proposed concept of ischemic territorial mismatch associated with PCA‐related collateral insufficiency in moyamoya disease [7].

Another notable feature of the present case was the marked hemispheric dissociation of the imaging findings, which provided an internal comparison of different hemodynamic conditions. In the left hemisphere, angiographic progression of ICA occlusive disease occurred during follow‐up; however, neither a FLAIR ivy sign nor recurrent ischemic symptoms developed. In contrast, the right hemisphere showed newly developed PCA occlusion accompanied by a prominent FLAIR ivy sign and recurrent ischemic events. We speculate that preserved extracranial‐to‐intracranial collateral flow through the previous STA–MCA bypass together with preserved PCA‐derived leptomeningeal collateral circulation maintained adequate hemodynamic compensation in the left hemisphere despite ICA progression. This hemispheric dissociation supports the interpretation that progressive PCA‐related collateral insufficiency, rather than ICA progression alone, was more closely associated with the newly developed FLAIR ivy sign and ischemic events in the right hemisphere.

The present findings may also provide insight into postoperative hemodynamic instability after revascularization surgery in moyamoya disease. In the present case, progressive PCA occlusion accompanied by a newly developed MCA‐dominant FLAIR ivy sign was associated with severe hemodynamic compromise, suggesting impaired cerebrovascular reserve within the affected MCA territory. Because impaired cerebrovascular reserve is a well‐recognized risk factor for postoperative cerebral hyperperfusion [8, 9], a newly developed MCA‐dominant FLAIR ivy sign may serve as an imaging marker of severe preoperative hemodynamic compromise.

Importantly, the present case demonstrates that postoperative hyperperfusion and acute infarction should be interpreted as distinct hemodynamic consequences despite sharing a common background of progressive PCA‐derived superficial leptomeningeal collateral insufficiency and impaired cerebrovascular reserve. Although the newly developed FLAIR ivy sign involved both the anterior and posterior MCA territories, acute infarction occurred predominantly in the posterior MCA territory, whereas postoperative hyperperfusion developed in the anterior MCA territory because the STA–MCA bypass recipient artery was intentionally selected from a frontal M4 cortical branch.

Taken together, the longitudinal and interhemispheric findings suggest that the clinical significance of PCA progression in moyamoya disease may extend beyond the anatomical PCA territory through loss of collateral support to the MCA territory. In this patient, the resulting hemodynamic disturbance was expressed differently across space and time, with posterior MCA infarction before surgery and focal anterior MCA hyperperfusion after bypass surgery.

Several limitations should be acknowledged. First, direct evaluation of cerebrovascular reserve using acetazolamide‐challenged perfusion studies was not performed. Second, detailed assessment of acute postoperative FLAIR changes associated with hyperperfusion was beyond the scope of this report. Finally, this report describes a single illustrative case, and further studies are required to clarify the generalizability of these longitudinal imaging findings in moyamoya disease.

## 4. Conclusion

This case demonstrates longitudinal changes in the FLAIR ivy sign in association with progressive PCA occlusion and subsequent revascularization. The within‐patient hemispheric comparison and angiographic findings suggest that deterioration of PCA‐derived collateral support, rather than ICA progression alone, was closely associated with the emergence of the ivy sign and ischemic events. The differing distributions of the ivy sign, posterior MCA infarction, and focal postoperative hyperperfusion further illustrate the spatially heterogeneous hemodynamic consequences of PCA‐related collateral insufficiency in moyamoya disease.

## Funding

No funding was received for this manuscript.

## Ethics Statement

This report is based on anonymized clinical imaging findings obtained during routine clinical care. According to Tohoku University Hospital policy, formal ethics committee review is not required for an anonymized individual case report of this nature. In accordance with the journal’s requirements and the ICMJE recommendations, potentially identifying information that was not essential to the scientific and educational purpose of this report was removed or generalized.

## Conflicts of Interest

The authors declare no conflicts of interest.

## Supporting information


**Supporting Information** Additional supporting information can be found online in the Supporting Information section.

## Data Availability

The data that support the findings of this study are available from the corresponding author upon reasonable request.
